# What Can Affect AOD–PM_2.5_ Association?

**DOI:** 10.1289/ehp.0901732

**Published:** 2010-03

**Authors:** Naresh Kumar

**Affiliations:** University of Iowa-Geography, Iowa City, Iowa, E-mail: naresh-kumar@uiowa.edu

Although satellite remote sensing has advanced significantly in recent years, there are inherent weaknesses in the use of this technology. The association between satellite-based aerosol optical depth (AOD_S_) and air pollution monitored on the ground can be influenced by a number of factors. In their article, Paciorek and Liu (2009) highlighted the weaknesses of AOD_S_ to predict the spatial distribution of fine particulate matter ≤ 2.5 μm in aerodynamic diameter (PM_2.5_). It is a timely article given the increasing importance of indirect methods, including satellite data, to estimate air quality because of scarce and ad hoc spatial–temporal coverage of air pollution monitored by federal regulatory methods. It is important that the robustness of these methods is evaluated, and Paciorek and Liu’s article is such an attempt. However, they failed to address the role of five major factors that can influence the AOD_S_–PM_2.5_ association. These factors include decomposition of AOD_S_ by aerosol types, mismatch in spatial–temporal resolution, collocation and integration of AOD_S_ and PM_2.5_ data, and control for spatial–temporal structure in the statistical model. Consequently, the weaknesses in Paciorek and Liu’s study lead me to question their findings.

The columnar measurement of AOD_S_ consists of aerosols generated by anthropogenic (human) sources (AOD_Sh_), such as emissions from industries and vehicles, and natural sources (AOD_Sn_), such as water vapor or dust in the air. AOD_Sn_ that constitutes a large fraction of AOD_S_ is influenced by moving large air masses and observes a strong spatial and temporal structure. The concentration of PM_2.5_, however, can vary significantly within short distances. Therefore, there is a significant mismatch in the magnitude and extent of spatial and temporal variability of AOD_Sn_ and AOD_Sh_; without an adequate control for AOD_Sn_, it is difficult to develop a reliable PM_2.5_ predictive model using AOD_S_ (Kumar et al. 2008).

Paciorek and Liu (2009) recognized that the spatial–temporal resolutions of AOD_S_ and PM_2.5_ they used were different, but they did not address how the mismatch in the spatial–temporal resolutions of these data can influence their association. The spatial resolutions of MISR (multiangle imaging spectroradiometer), MODIS (moderate resolution imaging spectroradiometer), and GEOS (geostationary operational environmental satellite) AOD were 17.6 km, 10 km, and 4 km, respectively, and PM_2.5_ data were point measurements aggregated across 24 hr. A recent study suggests the strength of the AOD_S_–PM_2.5_ association diminishes with the increase in time interval used for their aggregation (Kumar et al. 2007). It would have been useful for Paciorek and Liu (2009) to document the implications of the spatial–temporal resolutions and aggregation of AOD and PM_2.5_ (data they used) on their findings.

AOD_S_ retrieval and PM_2.5_ are not available on the same days: AOD_S_ retrieval is not possible on cloudy days, and PM_2.5_ data are recorded every third or sixth day. It seems that Paciorek and Liu (2009) averaged all AOD_S_ at 4-km pixel (i.e., 16 km^2^ area; monthly and yearly) and all PM_2.5_ (in the pixels where a monitoring station was situated). This could have resulted in a weak association between AOD_S_ and PM_2.5_, because there were systematic temporal gaps in both AOD_S_ and PM_2.5_ data sets. A reasonable approach to address this problem is to aggregate AOD_S_-PM_2.5_ data for those days only when both AOD_S_ and PM_2.5_ are available.

Paciorek and Liu’s method for aggregating 17.6-km and 10-km AOD_S_ to a 4-km pixel seems problematic. First, a radiative transfer model is used to retrieve AOD_S_ (Remer et al. 2006) which removes pixels with the upper 50% and lower 20% of the reflectance values. This removal can be systematic. For example, pixels with high reflectivity (such as buildings and roads) are more likely to be removed than the vegetated pixels (i.e., pixels under vegetation canopy). Thus, the centroid of a 10-km AOD_S_ pixel may not represent the AOD_S_ value for the entire 10-km area. Second, AOD_S_ registers a strong spatial–temporal autocorrelation. Thus, time–space kriging that utilizes large number of data points is appropriate for AOD_S_ aggregation rather than a single AOD_S_ value to avoid an area specific bias.

The robustness of AOD_S_ retrieval is evaluated by its comparison with the AOD recorded by sunphotometers at AERONET sites (AOD_A_) (NASA 2007). The spatial resolution at which AOD_S_ is retrieved and the spatial–temporal intervals within which these data are aggregated may directly influence its comparison with the AOD_A_ . This, in turn, can influence the association between AOD_S_ and PM_2.5_. Recent literature suggests that 1-km and 5-km AOD_S_ observe a significantly better association with PM_2.5_ monitored on the ground than the 10-km AOD_S_ (Kumar et al. 2007; Li et al. 2005). Therefore, the optimal spatial resolution of AOD_S_ retrieval and the optimal spatial and temporal intervals for aggregating these data are critically important for developing time–space resolved estimates of air quality with the aid of AOD_S_.

Because meteorologic conditions are largely influenced by the prevailing air masses and do not vary significantly within thousands of miles for a short period of time, the AOD_Sn_ component of AOD_S_ is likely to have a strong spatial–temporal structure. PM_2.5_ that constitutes particulate mass associated with anthropogenic factors, however, varies significantly within short distances from emission sources. Therefore, to develop a PM_2.5_ predictive model it is important that only AOD_Sh_ is used instead of AOD_Sn_. If such data are not available, an alternative is to indirectly control for AOD_Sn_ and its associated spatial–temporal structure. Otherwise the predicted PM_2.5_ surface is likely to have an unrealistic spatial trend, as reported by Paciorek and Liu (2009), as well as unrealistic temporal trends.

## References

[b1-ehp-118-a109] Kumar N, Chu A, Foster A (2007). An empirical relationship between PM_2.5_ and aerosol optical depth in Delhi Metropolitan. Atmos Environ.

[b2-ehp-118-a109] Kumar N, Chu A, Foster A (2008). Remote sensing of ambient particles in Delhi and its environs: estimation and validation. Int J Remote Sensing.

[b3-ehp-118-a109] Li C, Lau AK-H, Mao JT, Chu DA (2005). Retrieval, validation and application of 1-km resolution aerosol optical depth from MODIS data over Hong Kong. IEEE Trans Geosci Remote Sensing.

[b4-ehp-118-a109] NASA (2007). The AERONET (AErosol RObotic NETwork).

[b5-ehp-118-a109] Paciorek CJ, Liu Y (2009). Limitations of remotely sensed aerosol as a spatial proxy for fine particulate matter. Environ Health Perspect.

[b6-ehp-118-a109] Remer LA, Tanré D, Kaufman YJ, NASA (2006). Algorithm for remote sensing of tropospheric Aerosol from MODIS.

